# Cultural adaptation among doctors: findings from sister hospital programme in East Nusa Tenggara Province, Indonesia

**DOI:** 10.1186/1471-2458-14-S1-O5

**Published:** 2014-01-29

**Authors:** Atik Triratnawati

**Affiliations:** 1 Faculty of Cultural Science, Universitas Gadjah Mada, Yogyakarta 55281, Indonesia

## Background

East Nusa Tenggara (ENT) Province has adapted sister hospital (clinical contracting out) programme to decrease maternal and neonatal rate since 2010. The district hospitals from 11 regencies established partnership with larger hospitals from different places in Indonesia. Specialists from partner institutions (obstetrics and gynaecology, paediatrics, anaesthesia) who conducted health services transferred their knowledge, management skills and culture of work. Cultural adaptation is important process of the internalisation of the new values. The aims of this study were to identify and understand the process of cultural adaptation between two different types of hospitals in order to anticipate potential conflicts.

## Materials and methods

The study was conducted in 2010 - 2012. Observations and in-depth interviews were performed on 40 local physicians and specialists of 11 district hospitals.

## Results

The process of adaptation among doctors from two different types of hospitals in some cases may have increased the risk of conflict between them, due to the differences in culture of work. The local culture of work was the lack of discipline, full of hierarchy systems, and was relatively slower than the partner institutions. Culture shock that occurred among the local doctors was work culture, competency, self-esteem, and also differences in incentives. The clinical contracting out may influence inequality in relationship and subordinate. The local doctors felt that they were junior and unskilled. The potential conflict may occur if there is no mechanism of communication between them.

## Conclusions

Cultural adaptation is a long process that must be performed continuously, otherwise potential conflict may occur. Open forum and equal communication between local doctors and partners are essential.

